# Outer Surface Protein C Is a Dissemination-Facilitating Factor of *Borrelia burgdorferi* during Mammalian Infection

**DOI:** 10.1371/journal.pone.0015830

**Published:** 2010-12-31

**Authors:** Sunita V. Seemanapalli, Qilong Xu, Kristy McShan, Fang Ting Liang

**Affiliations:** Department of Pathobiological Sciences, Louisiana State University, Baton Rouge, Louisiana, United States of America; Singapore Immunology Network-A*STAR, Singapore

## Abstract

**Background:**

The Lyme disease spirochete *Borrelia burgdorferi* dramatically upregulates outer surface protein C (OspC) in response to fresh bloodmeal during transmission from the tick vector to a mammal, and abundantly produces the antigen during early infection. As OspC is an effective immune target, to evade the immune system *B. burgdorferi* downregulates the antigen once the anti-OspC humoral response has developed, suggesting an important role for OspC during early infection.

**Methodology/Principal Findings:**

In this study, a borrelial mutant producing an OspC antigen with a 5-amino-acid deletion was generated. The deletion didn't significantly increase the 50% infectious dose or reduce the tissue bacterial burden during infection of the murine host, indicating that the truncated OspC can effectively protect *B. burgdorferi* against innate elimination. However, the deletion greatly impaired the ability of *B. burgdorferi* to disseminate to remote tissues after inoculation into mice.

**Conclusions/Significance:**

The study indicates that OspC plays an important role in dissemination of *B. burgdorferi* during mammalian infection.

## Introduction

Coordinating production of outer surface proteins (Osps) is crucial for the pathogenic strategy of the Lyme disease spirochete, *Borrelia burgdorferi* [1]. In engorged and unfed ticks, *B. burgdorferi* abundantly produces OspA and OspB, but no OspC [2], [3]. In response to a fresh bloodmeal, the pathogen dramatically upregulates OspC and other RpoS-dependent genes to prepare itself for infection of mammals [4]–[6]. *B. burgdorferi* maintains high OspC synthesis during early mammalian infection [7]–[9]. However,OspC is not only a strong immunogen, but also an effective target of protective immunity; its production ultimately induces a robust humoral response that imposes tremendous pressure on the pathogen [10], [11]. To effectively evade the adaptive immune response and establish persistent infection, *B. burgdorferi* must downregulate *ospC* after the specific humoral response has developed [12], suggesting an early role for OspC in mammalian infection.

Inactivation of the *ospC* gene completely abolishes infectivity of *B. burgdorferi* [5]; however, the resulting mutant is able to persist in mammalian tissues once the initial requirement for OspC is overcome via introduction of an unstable *ospC* copy, which is eventually lost under the immune selection pressure during infection of immunocompetent mice, leading to a conclusion that OspC is required exclusively for initial mammalian infection [4], [13], [14]. However, this initial requirement for OspC can be overridden by either increasing expression of another Osp [15], or simply by adapting *ospC* mutants in mammalian hosts [16]. The nature of adaptation is to alter gene expression, and *B. burgdorferi* indeed undergoes dramatic changes in its surface lipoprotein expression during mammalian infection [8], [9]. Most notably, the downregulation of OspC in response to the development of the anti-OspC humoral response occurs concurrently with the upregulation of both VlsE and BBF01 [7]. Although remaining to be investigated, the host adaptation process most likely provides OspC-deficient spirochetes with both time and environment that enable the upregulation of other Osps, such as VlsE and BBF01, to occur during the course of disappearance of unstable *ospC* copies. The ability of an Osp to replace OspC in initial mammalian infection highlights a redundant function of the Osps, which is to protect *B. burgdorferi* against innate immune elimination [15]. However, increasing expression of an Osp fails to fully restore *ospC* mutants with expected dissemination ability [15], leading us to hypothesize that OspC is a dissemination-facilitating factor.

As described by some investigators, the protective and dissemination-promoting functions of OspC are more like two sides of the same coin [17], highlighting the challenge to dissect them. In this study, fortunately, we were able to generate a truncated OspC, which effectively protected *B. burgdorferi* against innate elimination, as measured by the 50% infectious dose (ID_50_) and tissue bacterial loads during murine infection, but failed to efficiently promote dissemination of *B. burgdorferi* to remote tissues. The study allowed us to conclude that OspC is a dissemination-facilitating factor of *B. burgdorferi*.

## Materials and Methods

### Previously generated strains and constructs used in the current study

The *B. burgdorferi* B31 clone 13A, the *ospC* mutant (Δ*ospC*), and the complemented clones Δ*ospC*/*FL/*1 and Δ*ospC*/*FL/*2 were generated previously [18]. The TA cloning vector pNCO1T was constructed in a previous study [19]. The shuttle vector pBBE22 was a gift from S. Norris [20]. The features of these clones and constructs are summarized in Table 1.

**Table 1:** Constructs and clones used in the study.

Construct or clone	Description	Source
pNCO1T	TA cloning vector	Reference [19]
pNCO1T-*ospC*	pNCO1T carrying *ospC* gene driven by its native promoter	This study
pNCO1T-*ospCnt5*	pNCO1T carrying *ospC* gene expressing N-terminus 5-AA deletion	This study
pNCO1T-*ospCnt10*	pNOC1T carrying *ospC* gene expressing N-terminus 10-AA deletion	This study
pBBE22	pBSV2 carrying a *bbe22* copy	Reference [20]
pBBE22-*ospCnt5*	pBBE22 carrying *ospC* gene expressing N-terminus 5-AA deletion	This study
pBBE22-*ospCnt10*	pBBE22 carrying *ospC* gene expressing N-terminus 10-AA deletion	This study
13A	*B. burgdorferi* B31 clone lacking plasmids lp25 and lp56	Reference [18]
Δ*ospC*	*ospC* mutant	Reference [18]
Δ*ospC*/*FL/*1	*ospC* mutant complemented with a wild-type *ospC* copy	Reference [18]
Δ*ospC*/*FL/*2	*ospC* mutant complemented with a wild-type *ospC* copy	Reference [18]
Δ*ospC*/Δ*Nt5/*1	*ospC* mutant expressing OspC with 5-AA deletion	This study
Δ*ospC*/Δ*Nt5/*2	*ospC* mutant expressing OspC with 5-AA deletion	This study
Δ*ospC*/Δ*Nt10/*1	*ospC* mutant expressing OspC with 10-AA deletion	This study
Δ*ospC*/Δ*Nt10/*2	*ospC* mutant expressing OspC with 10-AA deletion	This study

### Construction of pBBE22-*ospCnt5* and pBBE22-*ospCnt10*

**Figure 1:**
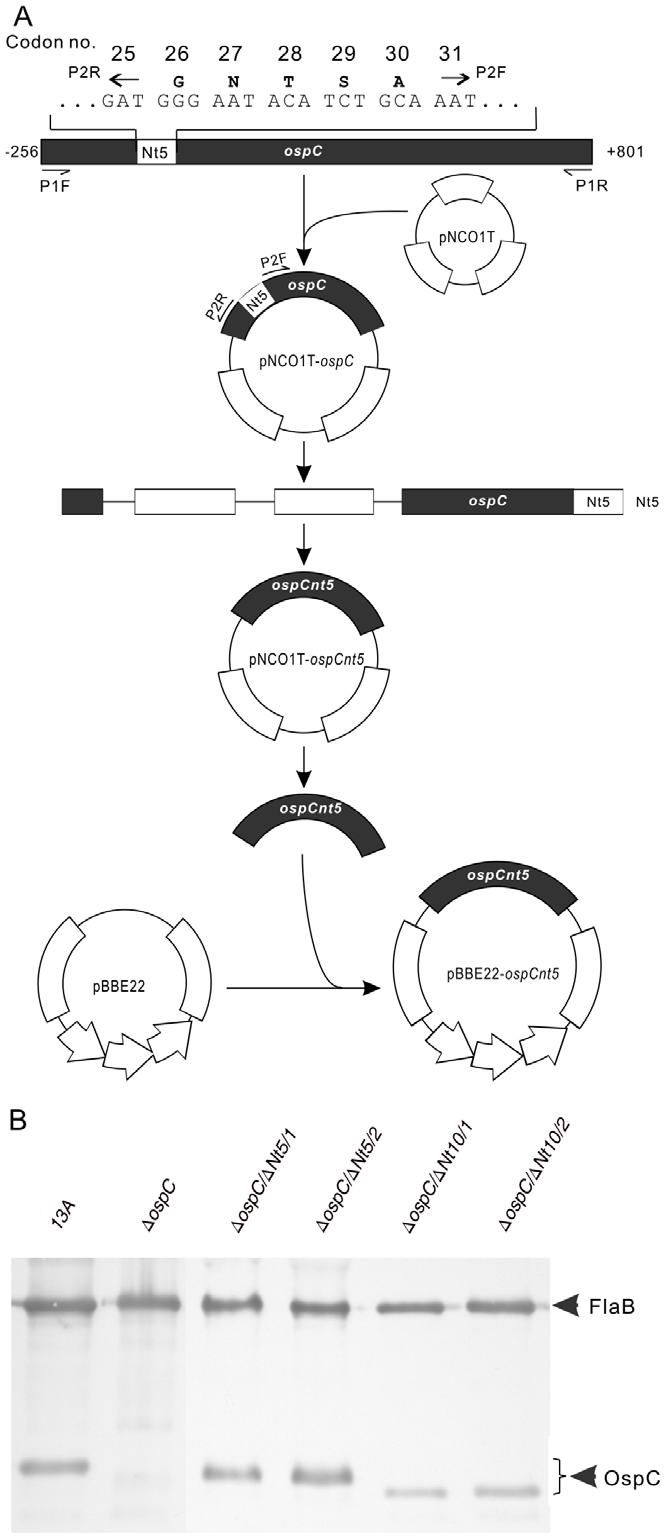
Generation of *B. burgdorferi* producing OspC with N-terminal 5-AA or 10-AA deletion. A) Construction of pBBE22-*ospCnt5* and pBBE22-*ospCnt10*. The five codons coding for the five amino acid residues (residues 26–30) and their adjacent codons are presented. The lipidation site, residue No. 19, is not shown. The amplification starting sites and directions of four primers, P1F, P1R, P2F and P2R, used for plasmid construction are also marked. The long bar represents the 1057-bp fragment, covering the entire *ospC*-coding region and down- (extending to +801 from the transcriptional start site) and up-stream sequences (extending to -256). The same strategy was used to construct pBBE22-*ospCnt10*. The detailed procedure was described in Materials and Methods. B) Restoration of OspC production. The parental clone 13A, Δ*ospC*, and the transformants Δ*ospC*/Δ*Nt5*/1, Δ*ospC*/Δ*Nt5*/2, Δ*ospC*/Δ*Nt10*/1 and Δ*ospC*/Δ*Nt10*/2 were verified for OspC expression by immunoblot probed with a mixture of FlaB and OspC MAbs.

As illustrated in Figure 1, to efficiently generate an N-terminus deletion, a 1057-bp fragment covering the *ospC* region and the up- and down-stream sequences was amplified from *B. burgdorferi* B31 genomic DNA by PCR using primers P1F and P1R (Table 2) and cloned into the TA cloning vector pNCO1T [19], creating an intermediate vector designated pNCO1T-*ospC*. One large amplicon was generated by inverse PCR, using pNCO1T-*ospC* as a template and primers P2F and P2R (Table 2). After digestion with *Sap*I and subsequent purification, the amplicon was circularized via ligation and then digested with *Bam*HI and *Xba*I to release *ospCnt5*. This fragment, encoding an OspC variant with N-terminal 5-amino acid (AA) deletion, was cloned into pBBE22 after the vector was digested with *Bam*HI and *Xba*I. The resulting construct was designated pBBE22-*ospCnt5*. The insert and its flanking regions within pBBE22 were sequenced to ensure the construct was as designed.

**Table 2:** Primers used in the study.

Primer	Sequence (5′—3′)^a^
P1F	TAGTTGGCTATATTGGGATCCAA
P1R	TTCCTCTAGAGAAGAGCTTAAAGTTAA
P2F	GACTGCTCTTCAGACAATTCTGCTGATGAGTCT
P2R	CGTACGCTCTTCAGTCTTTCCCAGAATTATTACAAG
P3F	ATGACGTCAAAGGGCCTA
P3R	CTGACGTCTTCCCCTGAATT

a The underlined sequences are restriction enzyme sites. P1F contains a *Bam*HI site; P1R has an *Xba*I site; P2F and P2R each have a *Sap*I site, and P3F and P3R have an *Aat*II site.

The same strategy was used to generate pBBE22-*ospCnt10*. Briefly, pNCO1T-*ospC* was amplified by using primers P3F and P3R (Table 2). The resultant PCR product was digested, circularized, re-digested, and finally cloned into pBBE22, as described above for constructing pBBE22-*ospCnt5*.

### Transformation of *B. burgdorferi* and selection of transformants

Constructs were electroporated into Δ*ospC*; resulting transformants were screened and analyzed for plasmid content as described previously [21]. Restoration of OspC production was verified using immunoblots probed with a mixture of FlaB and OspC MAbs, as described in an earlier study [18].

### Indirect immunofluorescence assay

*B. burgdorferi* was grown to late-log phase (10^8^ cells per ml) in BSK-H complete medium at 33°C. Approximately 2×10^7^ cells were harvested from 0.2 ml of culture by centrifugation at 10,000× *g* for 10 min, gently suspended in 100 µl PBS containing OspC MAb, and incubated for 1 hour at room temperature. After 2 washes with excess volumes of PBS by centrifugation at 16,000× *g* for 5 min, spirochetes were resuspended in 100 µl of PBS containing 1.0 µg of fluorescein isothiocyanate-conjugated goat anti-mouse IgG (Pierce Chemical Company, Rockford, IL), incubated for 1 hour, washed twice with PBS by centrifugation, resuspended in 50 µl PBS, applied to microscopic slides, and analyzed using Axio Imager (Carl Zeiss Microimaging, Inc., Thornwood, NY).

### Infection study

Groups of three C3H SCID mice (ages 4–6 week; provided by the Division of Laboratory Animal Medicine at Louisiana State University, Baton Rouge, LA) were inoculated with 10^5^ spirochetes. Mice were sacrificed 1 month post-inoculation; heart, tibiotarsal joint and skin specimens were harvested for spirochete culture in BSK-H complete medium. Cultures were examined for growing spirochetes under a darkfield microscope every week for up to 3 weeks. All animal procedures described here and below were approved by the Institutional Animal Care and Use Committee at Louisiana State University (LSU Protocol No. 07-019).

### Quick clearance study

Groups of four C3H SCID mice each received two intradermal/subcutaneous inoculations of 10^5^ spirochetes. The two inoculation sites were at least 2 cm apart. Two animals from each group were euthanized at 24 or 48 hours later; inoculation site skin specimens were harvested for spirochete culture in BSK-H medium.

### Determination of ID_50_ values

Spirochetes were grown to late-log phase (10^8^ cells per ml) in BSK-H complete medium at 33°C and 10-fold serially diluted with fresh medium. C3H SCID mice each received one single intradermal/subcutaneous injection of 100 µl of spirochetal suspension (containing 10^1^ to 10^4^ organisms). Mice were euthanized one month post-inoculation; heart, tibiotarsal joint, and skin (not from inoculation site) specimens were harvested for bacterial isolation. The ID_50_ value was calculated as described by Reed and Muench [22].

### Tissue spirochetal load analysis

C3H SCID mice each were intradermally/subcutaneously inoculated with 10^4^ spirochetes. Animals were sacrificed one month post-inoculation; heart, joint, and skin specimens were harvested for DNA extraction. DNA was quantified for the copy numbers of *flaB* and murine actin genes by quantitative PCR (qPCR) as described previously [21]. The tissue spirochete burden was expressed as *flaB* DNA copies per 10^6^ host cells (2×10^6^ actin DNA copies).

### Dissemination studies

C3H SCID or BALB/c wild-type mice each were given a single intradermal/subcutaneous injection of 10^3^ spirochetes and were euthanized at 1, 2, 3 and 4 weeks post-inoculation. Inoculation site and remote site skin, ear, heart and tiobiotarsal joint specimens were harvested for spirochete isolation as described previously [21]. Spirochetes were injected into the dermis of the chest, so the skin from the back was harvested as remote site.

### Statistical analysis

A two-tailed Student *t* test was used to calculate a *P* value for each two groups. A *P* value ≤0.05 was considered to be significant.

## Results

### Generation of *B. burgdorferi* producing OspC with either 5- or 10-AA deletion

The goal of the study was to dissect the dissemination-facilitating from protective function of OspC. Our strategy was to generate *ospC* mutants that can be protected from innate elimination but are unable to efficiently disseminate via mutagenizing the *ospC* gene. Based on X-ray analyses, OspC is a largely α-helical protein and may be a dimer with a characteristic central four-helical bundle formed by the association of the two longest helices, helices 1 and 5, from each subunit [23], [24]. Each subunit consists of five α-helices, two β-sheets and six loops, in addition to the amino (N-) terminal 23-AA linker and the carboxyl (C-) terminal 14-AA stretch. Neither the N-terminal linker nor the C-terminal stretch contributes to the core three-dimensional structure of OspC; instead, the α-helices 1 and 5 bring the two terminal regions in close proximity, where the lipoprotein is anchored to the bacterial outer membrane through lipidation of the first cysteine residue of the N-terminal linker. Within the core, several small secondary structures are present, including α-helix 4 (consisting of 8 AA), β-sheet 1 (5 AA) and 2 (5 AA). We first generated *B. burgdorferi* expressing OspC with each of these small units being deleted (data not shown). Unfortunately, none of the resulting mutants were infectious, suggesting that these sequences are critical for the functions of OspC.

Next, we focused on the N-terminal sequence. One role of this sequence is to carry the sorting signal, which determines the surface location of OspC. In *E. coli* the so-called “+2” rule determines the cellular location of a lipoprotein. However, the sorting signal of *B. burgdorferi* can extend up to the +4 position [25]. To create N-terminal deletions, which can be successfully sorted to the outer surface, we intended to generate mutations starting at the +7 position. The construct pBBE22-*ospCnt5* was generated as illustrated in Figure 1A.

The two constructs were electroporated into the *ospC* mutant, Δ*ospC*, which was generated and characterized in our previous study [18]. Because Δ*ospC* lacks lp25, the plasmid that carries the gene *bbe22* coding for a nicotinamidase essential for survival of *B. burgdorferi* in the mammalian environment, the recombinant plasmid pBBE22, which harbors a copy of *bbe22*, was used as the shuttle vector [20]. Six and seven transformants were obtained receiving each construct; plasmid analyses led to the selection of four clones, Δ*ospC*/Δ*Nt5*/1, Δ*ospC*/Δ*Nt5*/2, Δ*ospC*/Δ*Nt10*/1 and Δ*ospC*/Δ*Nt10*/2. These clones shared the same plasmid content as Δ*ospC*, which had lost lp25, lp5, lp21, lp56 and cp9 [18]. Expression of truncated OspC resulting from the introduced construct was confirmed by immunoblot analysis (Figure 1B).

### Neither the 5- nor the 10-AA deletion affects the surface location of OspC

Because the deletion was generated close to the N-terminus, which may harbor the sorting signal, it is important to show that the truncated OspC can be successfully sorted to the outer surface of *B. burgdorferi*. To this end, we used indirect immunofluorescence to locate truncated OspC. As shown in Fig. S1, both truncated proteins showed similar fluorescence patterns as the wild-type control, indicating that neither deletion influenced the cellular location of OspC.

### The 10- but not the 5-AA deletion abolishes infectivity

Groups of three C3H SCID mice were inoculated with 10^5^ spirochetes of the clone Δ*ospC*/Δ*Nt5/*1, Δ*ospC*/Δ*Nt5/*2, Δ*ospC*/Δ*Nt10/*1, or Δ*ospC*/Δ*Nt10/*2. As a control, mice were also inoculated with the clone Δ*ospC*/*FL/*1 or Δ*ospC*/*FL/*2. The clones Δ*ospC*/*FL*/1 and Δ*ospC*/*FL*/2 were generated via introduction of a full-length *ospC* gene carried by the shuttle vector pBBE22 into Δ*ospC* in our previous study [18]. All mice were sacrificed 1 month post-inoculation; heart, tibiotarsal joint and skin specimens were harvested for spirochete culture in BSK-H complete medium. As shown in Table 3, like the control clones, the Δ*ospC*/Δ*Nt5/*1 and Δ*ospC*/Δ*Nt5/*2 spirochetes were grown from each sample of all inoculated mice. In contrast, neither Δ*ospC*/Δ*Nt10/*1 nor Δ*ospC*/Δ*Nt10/*2 bacteria were recovered from any specimens, suggesting that the 10-AA deletion may completely abolish the functions of OspC.

**Table 3:** The 10-AA deletion abolishes infectivity of *B. burgdorferi* a.

	No. of cultures positive/total specimens examined						No. of mice infected/total mice inoculated
Clone	Heart		Joint		Skin	All sites	
Δ*ospC/FL/*1	3/3		3/3		3/3	9/9	3/3
Δ*ospC/FL/*2	3/3		3/3		3/3	9/9	3/3
Δ*ospC*/Δ*Nt5/*1	3/3		3/3		3/3	9/9	3/3
Δ*ospC*/Δ*Nt5/*2	3/3		3/3		3/3	9/9	3/3
Δ*ospC*/Δ*Nt10/*1	0/3		0/3		0/3	0/9	0/3
Δ*ospC*/Δ*Nt10/*2	0/3	0/3		0/3		0/9	0/3

a Groups of three C3H SCID mice were inoculated with 10^5^ spirochetes of the clone Δ*ospC*/*FL/*1, Δ*ospC*/*FL/*2, Δ*ospC*/Δ*Nt5/*1, Δ*ospC*/Δ*Nt5/*2, Δ*ospC*/Δ*Nt10/*1, or Δ*ospC*/Δ*Nt10/*2. Mice were sacrificed 1 month post-inoculation; heart, tibiotarsal joint and skin specimens were harvested for spirochete culture in BSK-H complete medium.

The Δ*ospC*/Δ*Nt10/*1 and Δ*ospC*/Δ*Nt10/*2 bacteria might remain at the inoculation sites without dissemination. To rule out this possibility, groups of four SCID mice each received two intradermal/subcutaneous inoculations of the clones Δ*ospC*/Δ*Nt10/*1, Δ*ospC*/Δ*Nt10*/2, Δ*ospC*/*FL*/1 or Δ*ospC*/*FL*/2. Animals were euthanized at 24 or 48 hours later. As a positive control, the Δ*ospC*/*FL/*1 and Δ*ospC*/*FL/*2 bacteria were consistently grown from each of the 16 inoculation sites from all eight inoculated mice (data not shown). In contrast, neither Δ*ospC*/Δ*Nt10/*1 nor Δ*ospC*/Δ*Nt10/*2 spirochetes were recovered from any of the 8 sites harvested at each time point, confirming that the 10-AA deletion completely destroys the functions of OspC and leads to quick clearance of spirochetes in the murine host.

### The 5-AA deletion does not affect the ID_50_ value in SCID mice

Because the ID_50_ value reflects the smallest number of organisms to sufficiently initiate an infection in 50% of inoculated individuals, it is the best measurable criterion to assess how a pathogen is protected against innate immune elimination, especially when this is measured in SCID mice. To examine whether the 5-AA deletion reduced the ability of OspC to protect *B. burgdorferi* against innate immunity, groups of three C3H SCID mice each received one single inoculation of 10^1^ to 10^4^ spirochetes of the clone Δ*ospC*/Δ*Nt5*/1, Δ*ospC*/Δ*Nt5*/2, Δ*ospC*/*FL*/1, or Δ*ospC*/*FL*/2. All animals were euthanized 1 month post-inoculation; heart, joint and skin specimens were cultured for spirochetes. The ID_50_ values of the clones Δ*ospC*/Δ*Nt5*/1 and Δ*ospC*/Δ*Nt5*/2 were 18 and 32 organisms, compared to 18 and 32 organisms determined for the clones Δ*ospC*/*FL*/1 and Δ*ospC*/*FL*/2, respectively (Table 4), indicating that the truncated OspC retains the full protective function as wild-type OspC.

**Table 4:** The 5-AA deletion does not significantly affect the ID_50_ value in immunodeficient mice a.

Clone and dose (no. of organisms)	No. of cultures positive/total no. of specimens examined				No. of mice infected/total no. of mice inoculated	ID_50_ (no. of organisms)
	Heart	Joint	Skin	All sites		
Δ*ospC/FL*/1						18
10^4^	3/3	3/3	3/3	9/9	3/3	
10^3^	3/3	3/3	3/3	9/9	3/3	
10^2^	3/3	3/3	3/3	9/9	3/3	
10^1^	1/3	1/3	1/3	3/9	1/3	
Δ*ospC*/*FL*/2						32
10^4^	3/3	3/3	3/3	9/9	3/3	
10^3^	3/3	3/3	3/3	9/9	3/3	
10^2^	3/3	3/3	3/3	9/9	3/3	
10^1^	0/3	0/3	0/3	0/9	0/3	
Δ*ospC*/Δ*Nt5*/1						18
10^4^	3/3	3/3	3/3	9/9	3/3	
10^3^	3/3	3/3	3/3	9/9	3/3	
10^2^	3/3	3/3	3/3	9/9	3/3	
10^1^	1/3	1/3	1/3	3/9	1/3	
Δ*ospC/*Δ*Nt5*/2						32
10^4^	3/3	3/3	3/3	9/9	3/3	
10^3^	3/3	3/3	3/3	9/9	3/3	
10^2^	2/3	2/3	2/3	6/9	2/3	
10^1^	1/3	1/3	1/3	3/9	1/3	

a The Δ*ospC*/*FL*/1, Δ*ospC*/*FL*/2, Δ*ospC*/Δ*Nt5*/1 and Δ*ospC/ΔNt5*/2 spirochetes were grown to late-log phase (10^8^ cells per ml) and 10-fold serially diluted with BSK-H medium. Approximately 100 µl of bacterial suspension was intradermally/subcutaneously inoculated into each C3H SCID mouse. Animals were sacrificed 1 month later; heart, tibiotarsal joint and skin specimens were harvested for bacterial isolation. The ID_50_ values were calculated by the method of Reed and Muench [22].

### The 5-AA deletion does not reduce spirochete burdens in joint or skin of SCID mice

**Figure 2:**
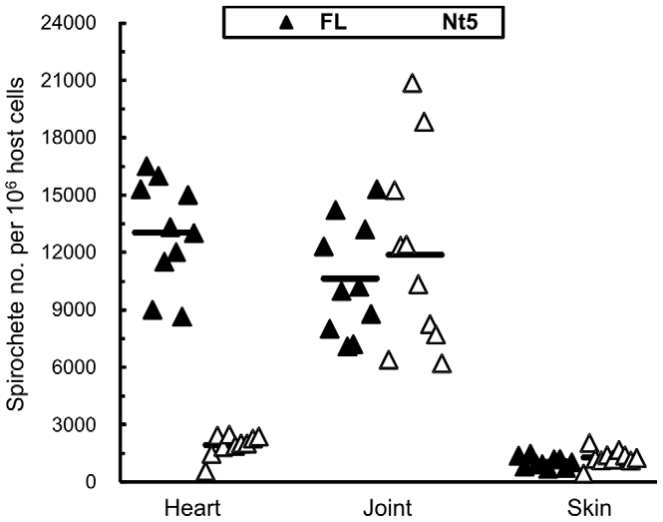
The 5-AA deletion does not reduce the ability of *B. burgdorferi* to colonize joint or skin tissue of SCID mice. Subgroups of five SCID mice were inoculated with 10^4^ spirochetes of the clone Δ*ospC*/*FL*/1, Δ*ospC*/*FL*2, Δ*ospC*/Δ*Nt5*/1 or Δ*ospC*/Δ*Nt5*/2, and euthanized a month later. DNA was prepared from heart, joint and skin specimens and analyzed for spirochete *flaB* and murine actin DNA copies by qPCR. Data are expressed as spirochete numbers per 10^6^ host cells and presented in two groups by combining the subgroups Δ*ospC*/*FL*/1 and Δ*ospC*/*FL*2, and Δ*ospC*/Δ*Nt5*/1 and Δ*ospC*/Δ*Nt5*/2.

Next, the tissue bacterial load was used to reflect whether the truncated OspC can effectively protect bacteria against innate elimination. To examine the influence of the 5-AA deletion on the tissue bacterial load, subgroups of five SCID mice each received a single intradermal/subcutaneous inoculation of 10^4^ spirochetes of the clone Δ*ospC*/Δ*Nt5*/1, Δ*ospC*/Δ*Nt5*/2, Δ*ospC*/*FL*/1, or Δ*ospC*/*FL*/2. In 10 mice that were inoculated with the Δ*ospC*/*FL*/1 or Δ*ospC*/*FL*/2, joint swelling evolved around 10 days post-inoculation and developed into severe arthritis a week later (data not shown). In the remaining mice, joint swelling did not become apparent until 3 weeks post-inoculation and slowly developed after then. All mice were euthanized 1 month post-inoculation; DNA was extracted from heart, joint and skin specimens and quantified for bacterial burden. Although the Δ*ospC*/Δ*Nt5* spirochete burden was 6.7-fold lower than that of the Δ*ospC*/*FL* (*P* = 2.83×10^−10^) in the heart tissue, there was no significant difference detected either in joint (*P* = 0.52) or skin tissue (*P* = 0.13) (Figure 2). The study indicated that the 5-AA deletion does not reduce the ability of OspC to protect *B. burgdorferi* against innate immunity.

### The 5-AA deletion leads to slowed dissemination during infection of SCID mice

The ID_50_ value was examined at one month post-inoculation. To determine whether the deletion caused deficiency in dissemination, we examined infection at different time points. Groups of 10 or 15 SCID mice each received a single intradermal/subcutaneous inoculation of the clone Δ*ospC*/*FL*/1 or Δ*ospC*/Δ*Nt5*/1. Five animals from each group were euthanized at 1-week intervals; inoculation site and remote site skin, ear, heart, and joint specimens were harvested for spirochete isolation. As a positive control, the Δ*ospC*/*FL*/1 bacteria were grown from all of the skin, joint and heart specimens but from none of the ear samples at first week; all sites became culture positive at 2 weeks after initial inoculation (Table 5). In contrast, the Δ*ospC*/Δ*Nt5*/1 spirochetes were grown only from inoculation sites at a week post-inoculation. Although the mutant disseminated to most of the joint, remote skin and heart specimens at 2 weeks, majority of ear tissues were not colonized until 3 weeks. These data indicated that OspC is important for efficient dissemination.

**Table 5:** The 5-AA deletion affects the ability of *B. burgdorferi* to disseminate to remote tissues in immunodeficient mice a.

Clone	No. of specimens positive/total specimens examined at post-inoculation weeks														
	1					2					3				
	I.S.	R.S.	Ear	Heart	Joint	I.S.	R.S.	Ear	Heart	Joint	I.S.	R.S.	Ear	Heart	Joint
Δ*ospC*/*FL*/1	5/5	5/5	0/5	5/5	5/5	5/5	5/5	5/5	5/5	5/5	ND^b^	ND	ND	ND	ND
Δ*ospC*/Δ*Nt5*/1	5/5	0/5	0/5	0/5	0/5	5/5	4/5	1/5	4/5	5/5	5/5	5/5	5/5	5/5	5/5

a Groups of 10 or 15 C3H SCID mice were inoculated with 10^3^ spirochetes of the clone Δ*ospC*/*FL/*1 or Δ*ospC*/Δ*Nt5/*1. Five animals from each group were euthanized at 1, 2, or 3 weeks post-inoculation; inoculation site (I.S.) and remote site (R.S.) skin, ear, heart, and tibiotarsal joint specimens were harvested for spirochete culture in BSK-H complete medium. The I.S. site was at the chest; therefore the R.S. site was at the back of the mice.

b Not determined.

### The 5-AA deletion causes more severely impaired dissemination during infection of immunocompetent mice

More severely impaired dissemination was noted during infection of immunocompetent mice. Groups of 24 BALB/c mice each received a single intradermal/subcutaneous inoculation of the clone Δ*ospC*/*FL*/1 or Δ*ospC*/Δ*Nt5*/1. Six animals from each group were euthanized at 1-week intervals; inoculation site and remote site skin, ear, heart, and joint specimens were harvested for spirochete isolation. As a positive control, the Δ*ospC*/*FL*/1 bacteria were grown from all of the remote tissues but the ear at first week; all sites became culture positive at 2 weeks after initial inoculation (Table 6). In contrast, the Δ*ospC*/Δ*Nt5*/1 spirochetes were not grown from joint tissues until 3 weeks, and even more significantly, no heart, remote skin or ear tissues became culture positive during the period of the 4-week study. These data further highlight that OspC is required for efficient dissemination.

**Table 6:** The 5-AA deletion severely impairs the ability of *B. burgdorferi* to disseminate in immunocompetent mice a.

Clone	No. of specimens positive/Total specimens examined at post-inoculation weeks																			
	1					2					3					4				
	I.S.	R.S.	Ear	Heart	Joint	I.S.	R.S.	Ear	Heart	Joint	I.S.	R.S.	Ear	Heart	Joint	I.S.	R.S.	Ear	Heart	Joint
*ΔospC*/*FL*/1	6/6	6/6	0/6	6/6	6/6	6/6	6/6	6/6	6/6	6/6	6/6	6/6	6/6	6/6	6/6	6/6	6/6	6/6	6/6	6/6
*ΔospC*/Δ*Nt5*/1	6/6	0/6	ND^b^	0/6	1/6	1/6	0/6	0/6	0/6	0/6	2/6	0/6	0/6	0/6	6/6	6/6	0/6	0/6	0/6	6/6

a Groups of 24 BALB/c mice each received a single intradermal/subcutaneous injection of 10^5^ spirochetes of the clone Δ*ospC*/*FL*/1 or Δ*ospC*/Δ*Nt5/*1. Six animals from each group were euthanized at 1, 2, 3, or 4 weeks post-inoculation; inoculation site (I.S.) and remote site (R.S.) skin, ear, heart, and tibiotarsal joint specimens were harvested for spirochete culture in BSK-H complete medium. The I.S. site was at the chest; therefore the R.S. site was at the back of the mice.

b Not determined.

## Discussion

Like typical Gram-negative bacteria, *B. burgdorferi* possesses inner and outer membranes, between which is a periplasmic space [26], [27]. Gram-negative pathogens make a thick LPS coat to provide a broad array of crucial protection [28]. However, *B. burgdorferi* does not produce any LPS, but instead, abundantly expresses lipoproteins and anchors them to the outer membranous surface through lipidation [26], [29], [30]. This unique surface antigenic structure may facilitate the host-pathogen interactions and potentially contribute to the pathogenic strategy of *B. burgdorferi*. As protein is a direct product of gene expression, regulation of surface antigen expression can quickly result in modification of the surface antigenic architecture. *B. burgdorferi* indeed takes advantage of this and vigorously modifies its surface lipoprotein expression to constantly reformulate its overall surface antigenic architecture during its enzootic life cycle traveling between the tick vector and a mammal, and during the course of mammalian infection. It abundantly expresses OspA and OspB in the unfed tick, because both may play an important role in the tick vector [2], [3], [31], [32]. A fresh bloodmeal induces the down-regulation of OspA/B and the up-regulation of OspC and other Osps, a process that prepares *B. burgdorferi* for infection of mammals [4]–[6], [33]. Abundant OspC expression ultimately induces a robust early humoral response that imposes tremendous pressure on the pathogen [10], [12]. To evade the specific humoral response and cause persistent infection, *B. burgdorferi* down-regulates OspC and dramatically upregulates other surface lipoproteins, including VlsE and BBF01 [7]–[9], [34]. This well-defined *ospC* expression pattern suggests an early role in mammalian infection.

Recent studies showed that this initial requirement for OspC can be readily overridden by either increasing expression of another Osp [15], or by simply adapting *ospC* mutants in mammalian hosts [16]. Clearly, the adaptation process provides *B. burgdorferi* with both the environment and time to increase expression of other Osps, such as VlsE and BBF01, when OspC production is being reduced in response to development of an anti-OspC humoral response [7]. Taken together, these studies indicate that increasing expression of an Osp(s), either via genetic modification or adaptation, can overcome the initial requirement for OspC, leading us to hypothesize that the borrelial lipoproteins may have a common function, which is to protect *B. burgdorferi* from innate elimination. To survive in the mammalian host, *B. burgdorferi* must maintain a certain level of surface lipoprotein production. During early infection, *B. burgdorferi* abundantly produces OspC. In addition to this common function, the specific function of OspC is to facilitate dissemination, which can be only partially replaced by another Osp. In the current study, we were able to separate protective and dissemination-facilitating functions as we successfully generated a truncated OspC, which can effectively protect *B. burgdorferi* against innate elimination but fails to make spirochetes to disseminate as efficiently as the wild-type control.

Our previous study showed that increasing production of one of four randomly chosen surface lipoproteins can replace OspC to protect *B. burgdorferi* from innate elimination, suggesting that most surface lipoproteins, if not all, can substitute OspC for this function. However, what strikes us is that OspC itself, after mutation, fails to protect *B. burgdorferi* against innate elimination. Although the 5-AA deletion retains the full protective function of OspC, several other deletions, including that of the N-terminal 10-AA, α-helix 4 (8 AA), and β-sheet 1 (5 AA) and 2 (5 AA), completely destroys its protective function (Seemanapalli and Liang, unpublished data). This leads us to hypothesize that Osps may play a common structural role that is required for the protective function of the borrelial surface lipoproteins. Some deletions on OspC may cause this special structure to collapse and consequently abolish the protective function.

After inoculated into murine skin, *B. burgdorferi* must first be able to evade initial innate immune elimination and gain a foothold. This ability is best reflected by the ID_50_ value because this criterion measures the smallest number of organisms that is sufficient to escape initial innate elimination and establish an infection. In spite of large inocula, OspC-deficient spirochetes are quickly cleared after inoculation into murine skin [14], [15]. Production of OspC with 5-AA deletion fully restored OspC-deficient *B. burgdorferi* with ID_50_ values similar to the control, indicating that the truncated molecule retains the full protective function of OspC.

Although tissue colonization is an extremely complicated event carried out by an extracellular bacterial pathogen during infection and can be affected by many factors, one can envision the ability of the pathogen to evade immune clearance should be one of the major determinants. *B. burgdorferi* producing OspC with 5-AA deletion was able to generate similar bacterial loads in both joint and skin tissues of SCID mice as the control, indicating that the truncated OspC can effectively protect the pathogen against innate immune clearance at least in these tissues. Because many other determinants, such as the interactions of host cells and extracellular matrices with the pathogen mediated by its surface antigens, may significantly influence tissue colonization, the lower Δ*ospC*/Δ*Nt5* spirochete load in the heart does not necessarily suggest that the truncated OspC cannot effectively provide protection against innate immune elimination. Alternatively, OspC may be an important factor contributing to the ability of *B. burgdorferi* to colonize the heart tissue. This explanation is also supported by previous studies showing that treatment with OspC antibody shuts off *ospC* expression and more effectively reduces the bacterial load in the heart than other tissues [7], [11] and that OspC-deficient spirochetes with increased expression of another Osp register reduced bacterial loads in the heart but not other tissues of SCID mice [15].

Both ID_50_ and tissue bacterial load data demonstrated that the 5-AA deletion does not affect the protective function of OspC, allowing us to dissect the protective from dissemination-facilitating functions. *B. burgdorferi* producing OspC with 5-AA deletion disseminated to remote tissues, especially to the heart and ear at a remarkably slow pace in immunodeficient mice. In immunocompetent mice, the defect in dissemination caused by deletion was much more severe. Four weeks after inoculation, the mutant was not able to colonize heart or ear tissues, in contrast to the control, which disseminated to all tissues examined within 2 weeks. The study also highlights the importance of quick dissemination during infection of immunocompetent animals. As infection induces immune responses, if *B. burgdorferi* fails to colonize remote tissues and establish a systemic infection before effective immune responses develop, its infectivity potential would be severely impaired.

## Supporting Information

Figure S1 **Deletion of N-terminus 5-AA or 10-AA does not affect the surface location of OspC.** The *ΔospC/FL*/1, *ΔospC/ΔNt5*/1 or *ΔospC/ΔNt10*/1 spirochetes were grown to late log phase (10^8^ per ml) in BSK-H medium at 33°C. Spirochetes were incubated with OspC MAb, washed in PBS, probed with FITC-conjugated goat anti-mouse antibody, washed again before being placed onto slides. Differential interference contrast (left panel) and immunofluorescence images (right panel) were taken from the same field. Imagines were taken at ×400 magnification.
(PDF)
