# Supplementary material for: Outer Surface Protein C Is a Dissemination-Facilitating Factor of Borrelia burgdorferi during Mammalian Infection
Source: PLoS One. 2010 Dec 31;5(12):e15830. doi: 10.1371/journal.pone.0015830 (PMC3013124; doi:10.1371/journal.pone.0015830)
Supplement: Figure S1 — Deletion of N-terminus 5-AA or 10-AA does not affect the surface location of OspC. The ΔospC/FL/1, ΔospC/ΔNt5/1 or ΔospC/ΔNt10/1 spirochetes were grown to late log phase (108 per ml) in BSK-H medium at 33°C. Spirochetes were incubated with OspC MAb, washed in PBS, probed with FITC-conjugated goat anti-mouse antibody, washed again before being placed onto slides. Differential interference contrast (left panel) and immunofluorescence images (right panel) were taken from the same field. Imagines were taken at ×400 magnification. (PDF) [file pone.0015830.s001.pdf]

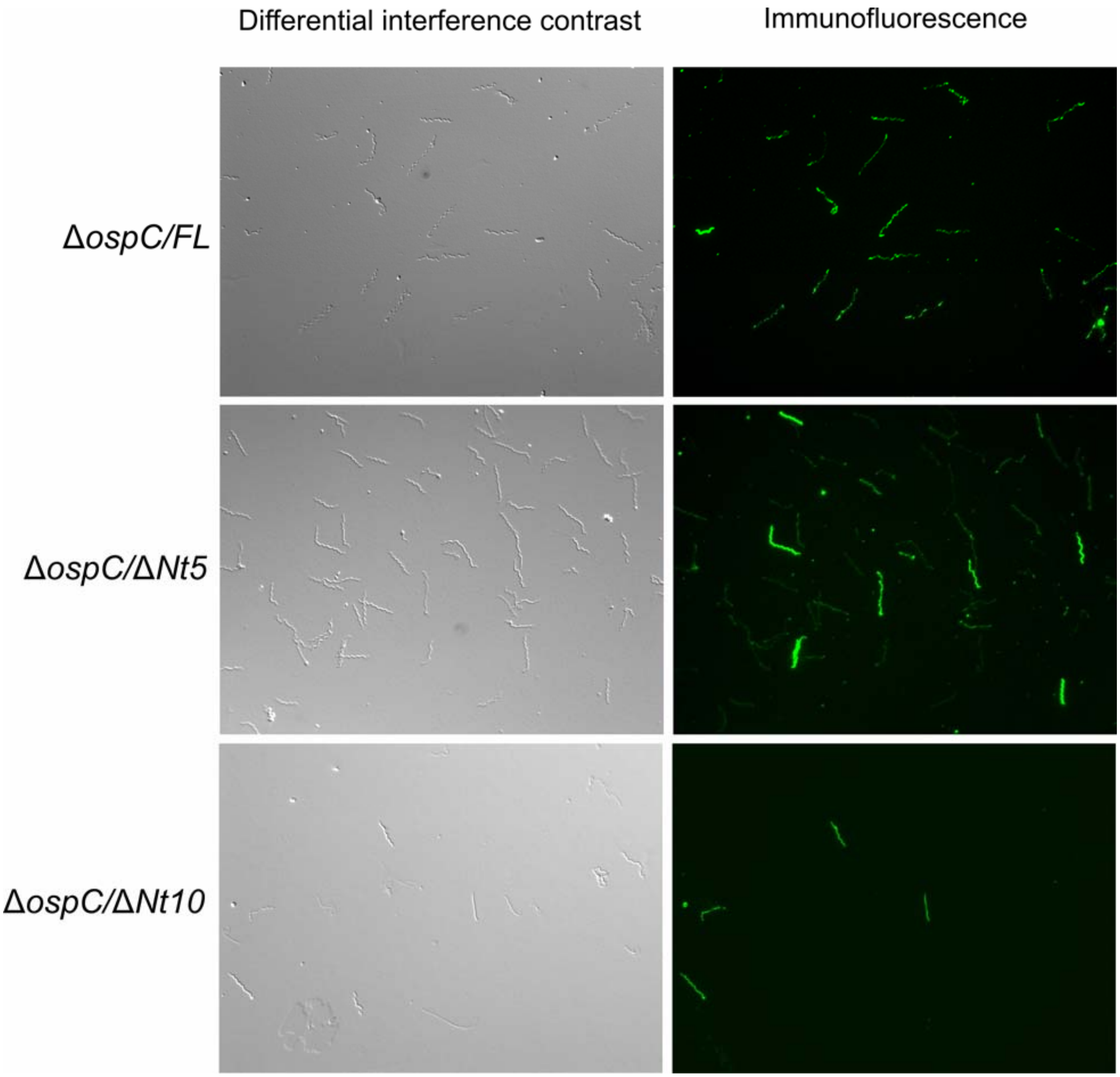
Differential interference contrast
Immunofluorescence
Δ*ospC/FL*
Δ*ospC/*Δ*Nt5*
Δ*ospC/*Δ*Nt10*
